# Supplementary material for: Molecular Tools to Infer Resistance-Breaking Abilities of Rice Yellow Mottle Virus Isolates
Source: Viruses. 2023 Apr 13;15(4):959. doi: 10.3390/v15040959 (PMC10144094; doi:10.3390/v15040959)
Supplement: Supplementary file 1 [file viruses-15-00959-s001.zip › viruses-2305375-supplementary/Figure S1.pptx]

## Slide 1
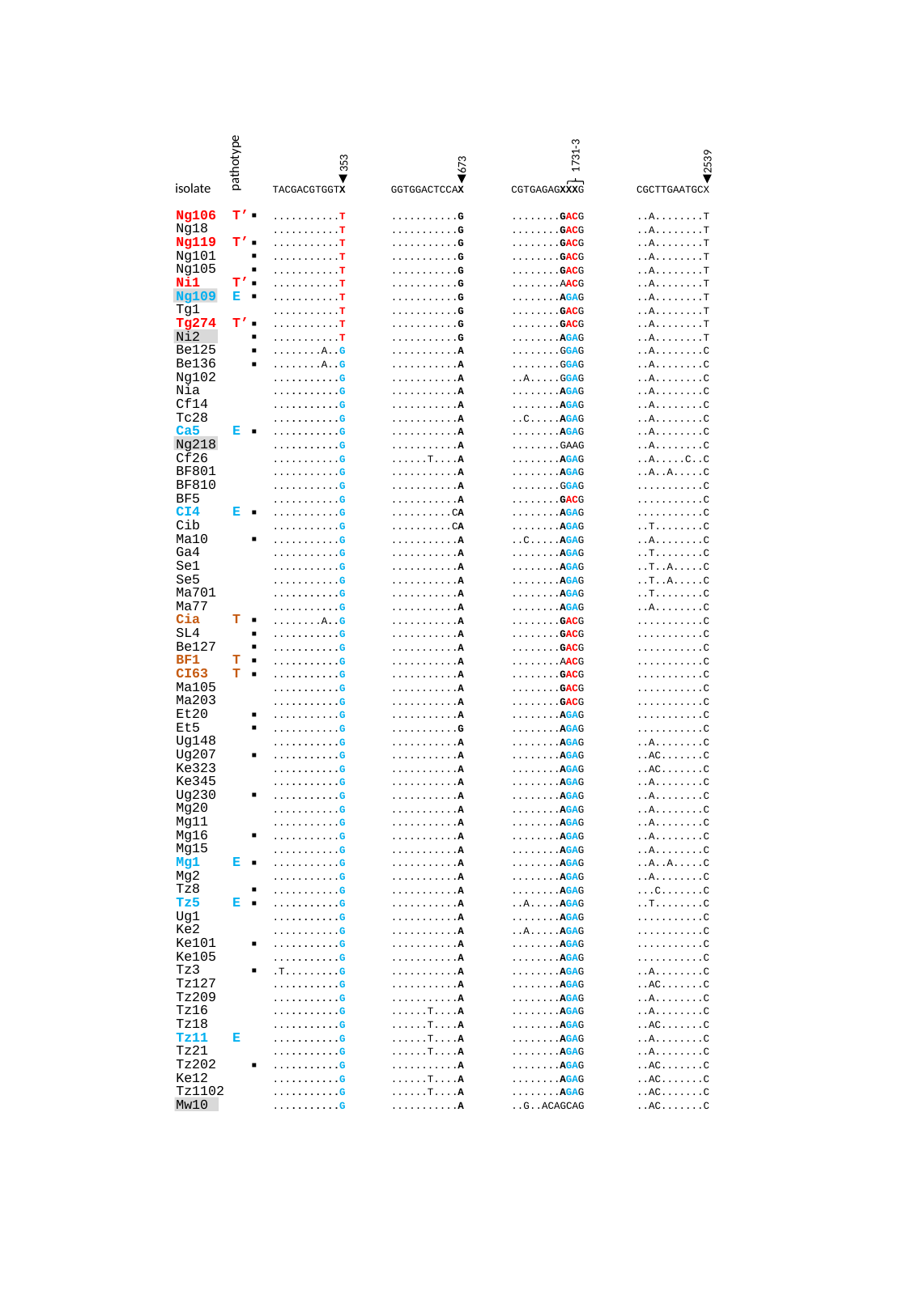

1731-3
673
2539
353
pathotype
isolate
TACGACGTGGTX
...........T
...........T
...........T
...........T
...........T
...........T
...........T
...........T
...........T
...........T
........A..G
........A..G
...........G
...........G
...........G
...........G
...........G
...........G
...........G
...........G
...........G
...........G
...........G
...........G
...........G
...........G
...........G
...........G
...........G
...........G
........A..G
...........G
...........G
...........G
...........G
...........G
...........G
...........G
...........G
...........G
...........G
...........G
...........G
...........G
...........G
...........G
...........G
...........G
...........G
...........G
...........G
...........G
...........G
...........G
...........G
...........G
.T.........G
...........G
...........G
...........G
...........G
...........G
...........G
...........G
...........G
...........G
...........G
GGTGGACTCCAX
...........G
...........G
...........G
...........G
...........G
...........G
...........G
...........G
...........G
...........G
...........A
...........A
...........A
...........A
...........A
...........A
...........A
...........A
......T....A
...........A
...........A
...........A
..........CA
..........CA
...........A
...........A
...........A
...........A
...........A
...........A
...........A
...........A
...........A
...........A
...........A
...........A
...........A
...........A
...........G
...........A
...........A
...........A
...........A
...........A
...........A
...........A
...........A
...........A
...........A
...........A
...........A
...........A
...........A
...........A
...........A
...........A
...........A
...........A
...........A
......T....A
......T....A
......T....A
......T....A
...........A
......T....A
......T....A
...........A
CGTGAGAGXXXG
........GACG
........GACG
........GACG
........GACG
........GACG
........AACG
........AGAG
........GACG
........GACG
........AGAG
........GGAG
........GGAG ..A.....GGAG
........AGAG
........AGAG
..C.....AGAG
........AGAG
........GAAG
........AGAG
........AGAG
........GGAG
........GACG
........AGAG
........AGAG
..C.....AGAG
........AGAG
........AGAG
........AGAG
........AGAG
........AGAG
........GACG
........GACG
........GACG
........AACG
........GACG
........GACG
........GACG
........AGAG
........AGAG
........AGAG
........AGAG
........AGAG
........AGAG
........AGAG
........AGAG
........AGAG
........AGAG
........AGAG
........AGAG
........AGAG
........AGAG
..A.....AGAG
........AGAG
..A.....AGAG
........AGAG
........AGAG
........AGAG
........AGAG
........AGAG
........AGAG
........AGAG
........AGAG
........AGAG
........AGAG
........AGAG
........AGAG
..G..ACAGCAG
CGCTTGAATGCX
..A........T
..A........T
..A........T
..A........T
..A........T
..A........T
..A........T
..A........T
..A........T
..A........T
..A........C
..A........C
..A........C
..A........C
..A........C
..A........C
..A........C
..A........C
..A.....C..C
..A..A.....C
...........C
...........C
...........C
..T........C
..A........C
..T........C
..T..A.....C
..T..A.....C
..T........C
..A........C
...........C
...........C
...........C
...........C
...........C
...........C
...........C
...........C
...........C
..A........C
..AC.......C
..AC.......C
..A........C
..A........C
..A........C
..A........C
..A........C
..A........C
..A..A.....C
..A........C
...C.......C
..T........C
...........C
...........C
...........C
...........C
..A........C
..AC.......C
..A........C
..A........C
..AC.......C
..A........C
..A........C
..AC.......C
..AC.......C
..AC.......C
..AC.......C
Ng106 T’
Ng18
Ng119 T’
Ng101
Ng105
Ni1 T’
Ng109 E
Tg1
Tg274 T’
Ni2
Be125
Be136
Ng102
Nia
Cf14
Tc28
Ca5 E
Ng218
Cf26
BF801
BF810
BF5
CI4 E
Cib
Ma10
Ga4
Se1
Se5
Ma701
Ma77
Cia T
SL4
Be127
BF1 T
CI63 T
Ma105
Ma203
Et20
Et5
Ug148
Ug207
Ke323
Ke345
Ug230
Mg20
Mg11
Mg16
Mg15
Mg1 E
Mg2
Tz8
Tz5 E
Ug1
Ke2
Ke101
Ke105
Tz3
Tz127
Tz209
Tz16
Tz18
Tz11 E
Tz21
Tz202
Ke12
Tz1102
Mw10
