# Supplementary figures and images for: Molecular Tools to Infer Resistance-Breaking Abilities of Rice Yellow Mottle Virus Isolates
Source: Viruses. 2023 Apr 13;15(4):959. doi: 10.3390/v15040959 (PMC10144094; doi:10.3390/v15040959)

## Slide 1
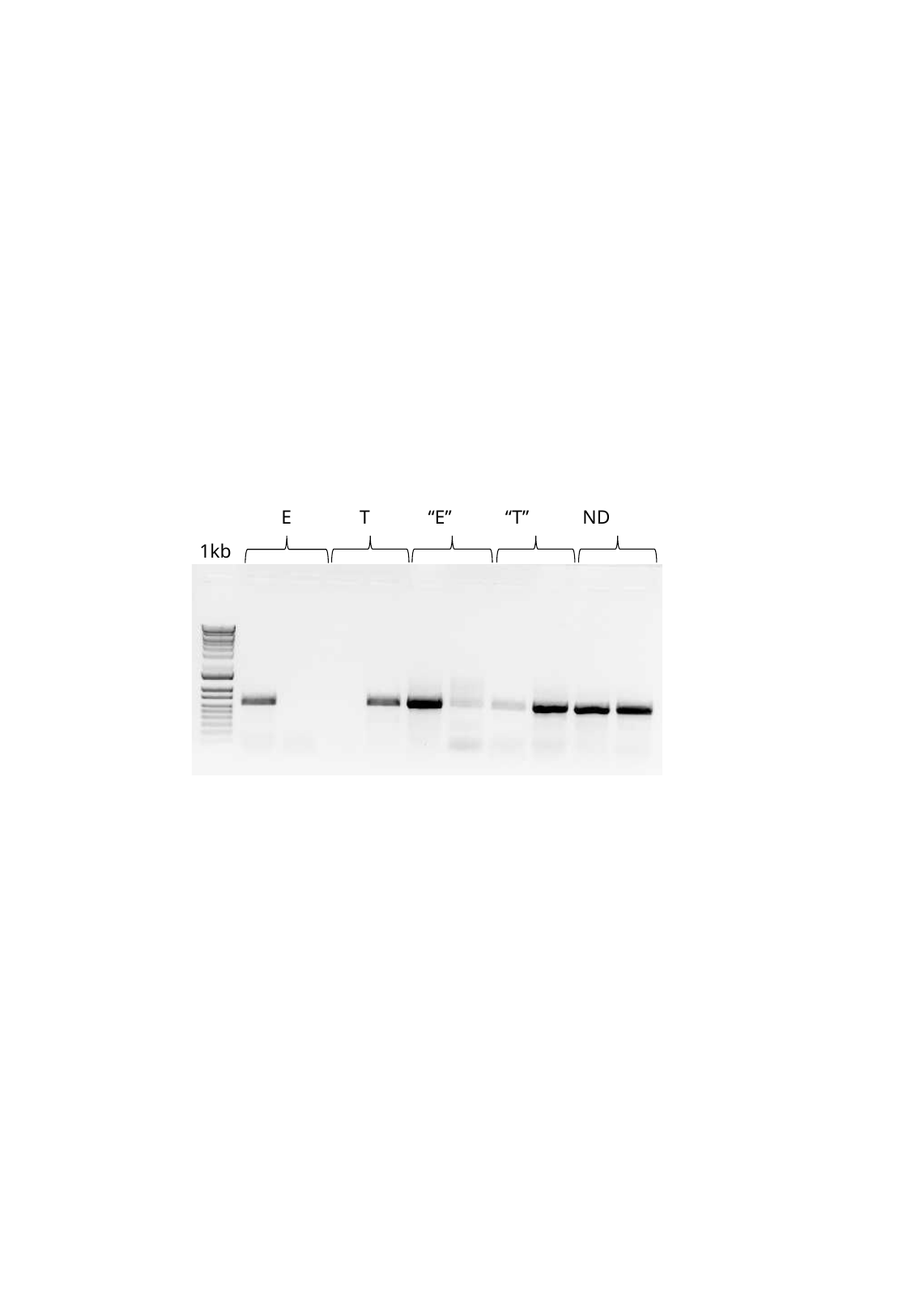

E T “E” “T” ND
 1kb

Supplement: Supplementary file 1 [file viruses-15-00959-s001.zip › viruses-2305375-supplementary/Figure S2.pptx]
